# Supplementary material for: Physical and functional interaction between A20 and ATG16L1-WD40 domain in the control of intestinal homeostasis
Source: Nat Commun. 2019 Apr 23;10:1834. doi: 10.1038/s41467-019-09667-z (PMC6478926; doi:10.1038/s41467-019-09667-z)
Supplement: Supplementary file 1 — Supplementary Information [file 41467_2019_9667_MOESM1_ESM.pdf]

Supplementary Information

Physical and functional interaction between A20 and ATG16L1-WD40 domain in the control of intestinal homeostasis

Slowicka *et al.*

Supplementary Figure 1

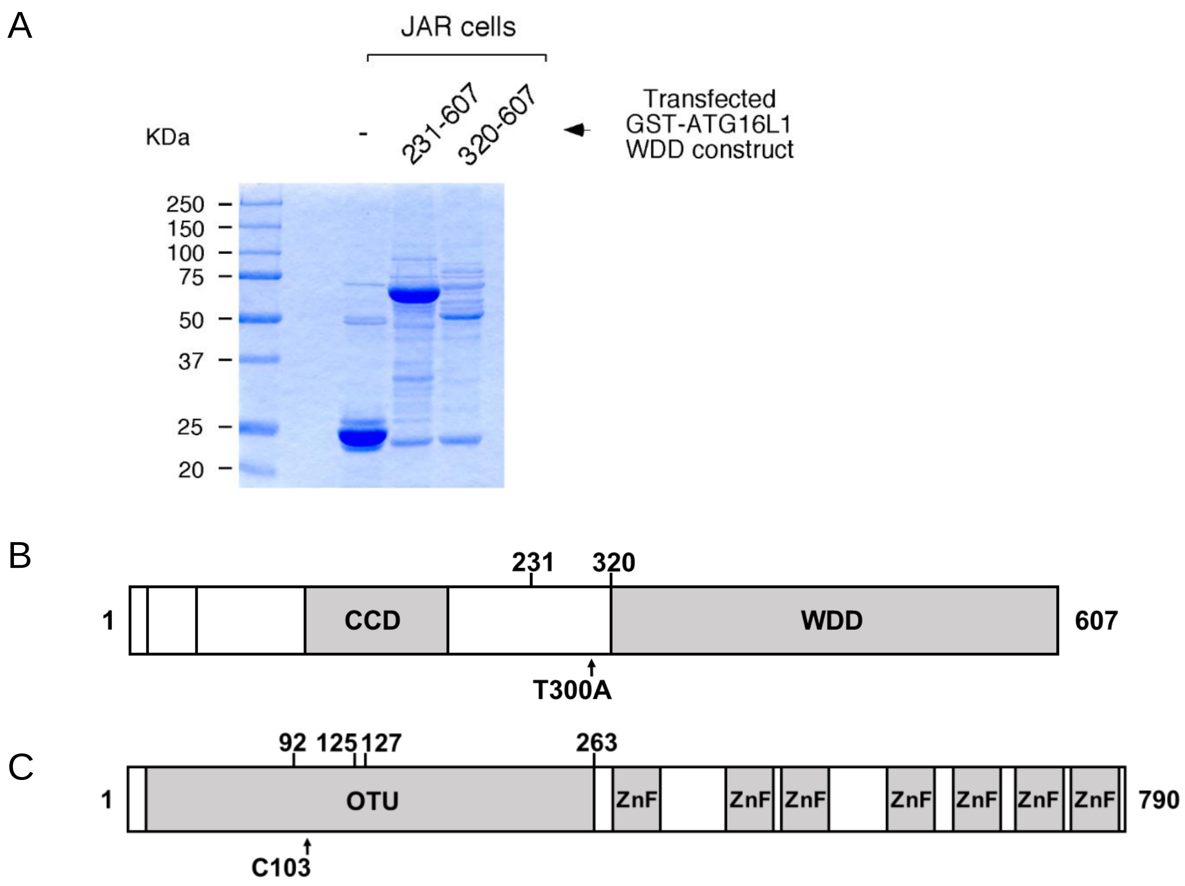

Supplementary Figure 1. Superior stability of GST-ATG16L1 231-607 compared to GST-ATG16L1 320-607 upon expression in JAR cells. **(A)** Cells were transfected with mammalian expression plasmids encoding the indicated constructs (5 x 6 cm plates per construct). 36 h post-transfection, cells were lysed and the resulting lysates incubated with agarose-GST beads for immunoprecipitation of the fusion proteins. After two washes, beads were resuspended in 2x RSB and boiled. Half of the final supernatant (equivalent to 3 x 6 cm plates per construct) was resolved in a 10% polyacrylamide gel and stained with Coomassie solution. Shown is a picture of the stained gel indicating the transfected constructs in each case. **(B)** Domain structure of human ATG16L1 with the coiled-coil domain (CCD) and the N-terminal WD40 domain (WDD). T300A represents the Crohn's Disease susceptibility polymorphism. **(C)** Domain structure of human A20. The N-terminal OTU domain is essential for deubiquitinating (DUB) activity. C103 represents the catalytic cysteine residue. The C-terminal region of A20 contains seven zinc fingers (ZnF).

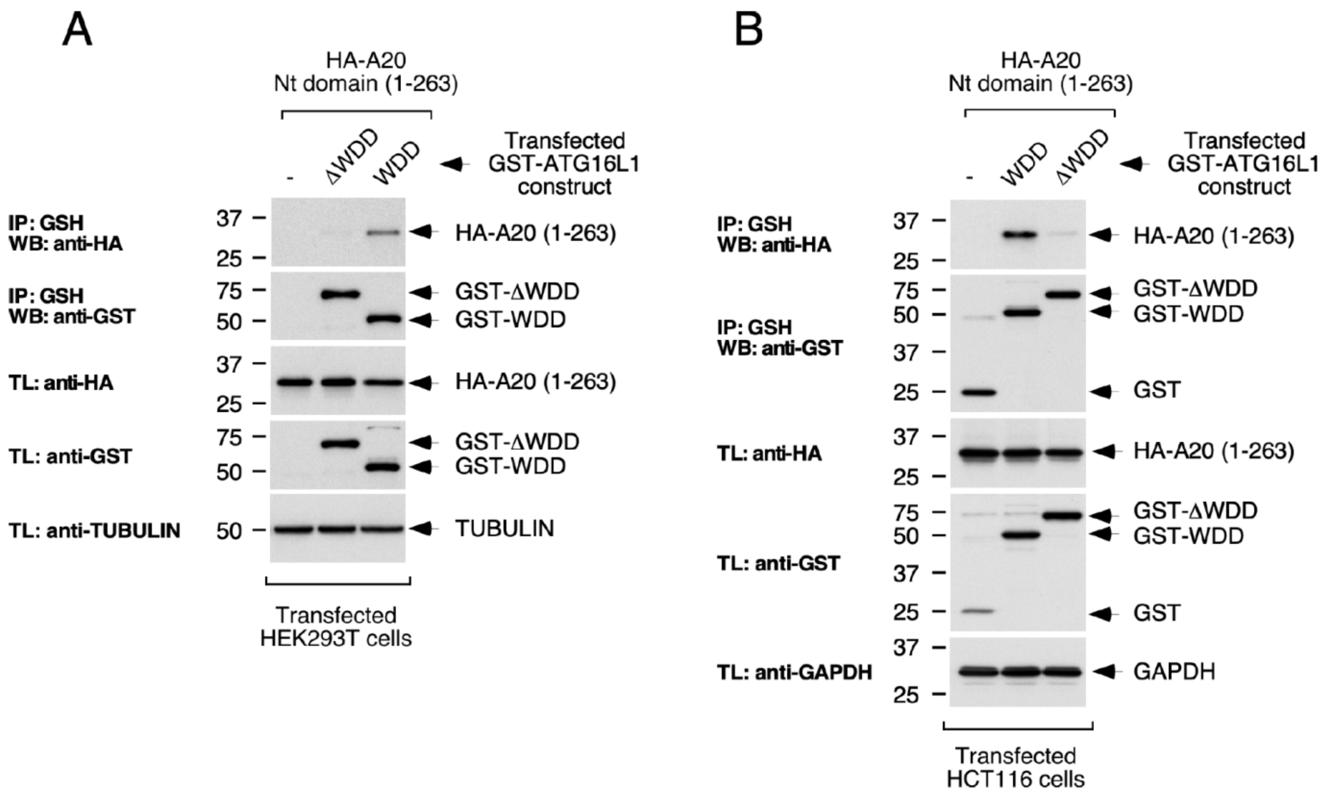

Supplementary Figure 2. The WDD of ATG16L1 (residues 320-607) interacts with A20 in both HEK293T and intestinal HCT116 cells. (**A-B**) A20-1-263 specifically co-precipitates with GST-WDD but not with GST-ΔWDD in both HEK293T cells (A) and HCT116 cells (B). The displayed co-immunoprecipitation assays were done as in Fig. 2.

Supplementary Figure 3

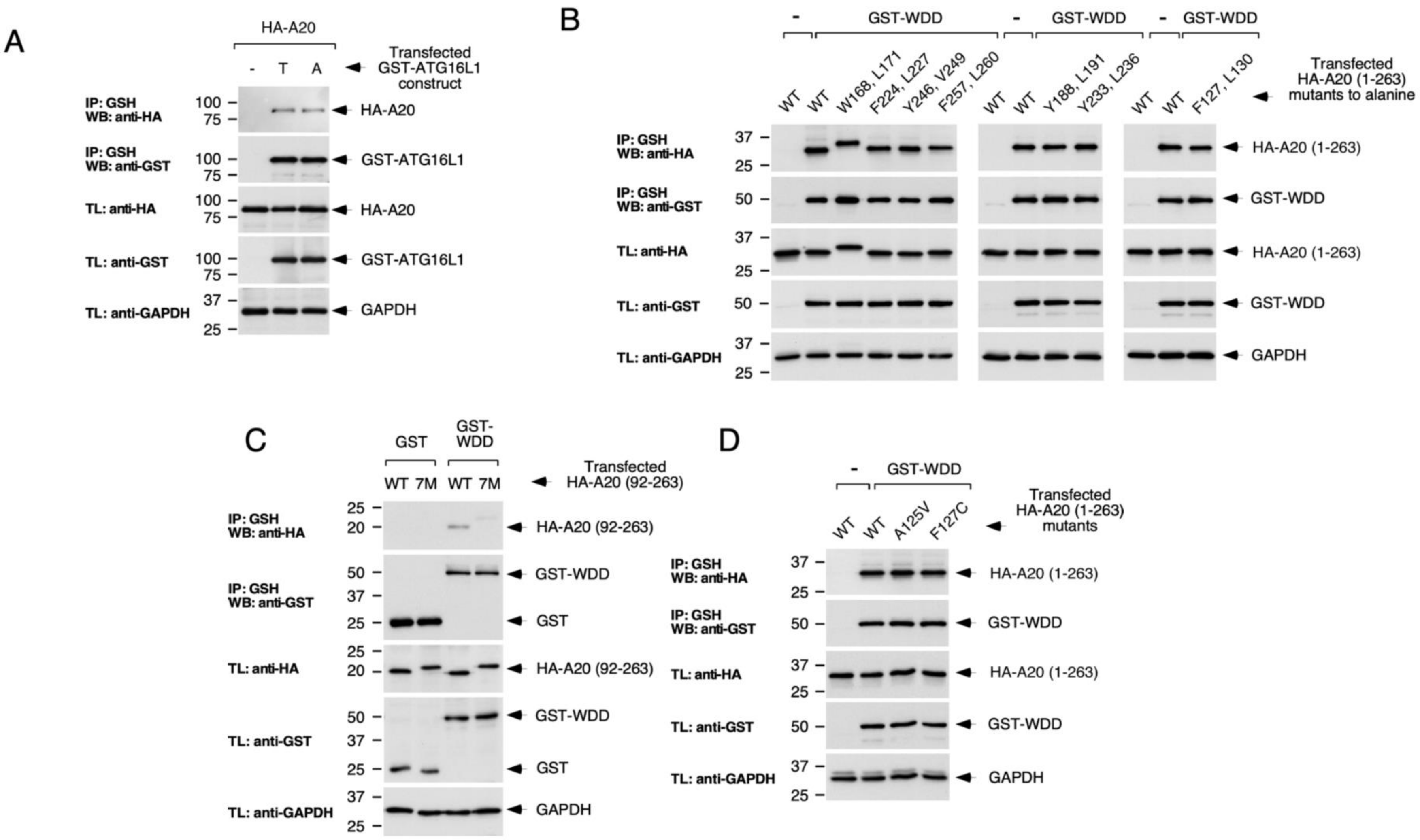

Supplementary Figure 3. (A) The Crohn's disease risk mutation T300A does not alter the ability of ATG16L1 to co-precipitate with A20. **(B)** Individual mutation of candidate WDD-binding motifs present in A20 92-263 region does not impair the interaction between A20 1-263 and the WDD. **(C)** Simultaneous mutation of the 7 candidate WDD-binding motifs (7M) prevents binding between A20 92-263 and the WDD. **(D)** Mutations A125V and F127C in A20 do not influence the interaction between A20 1-263 and the WDD. All panels show co-immunoprecipitation assays carried out with the indicated constructs as in Fig. 2.

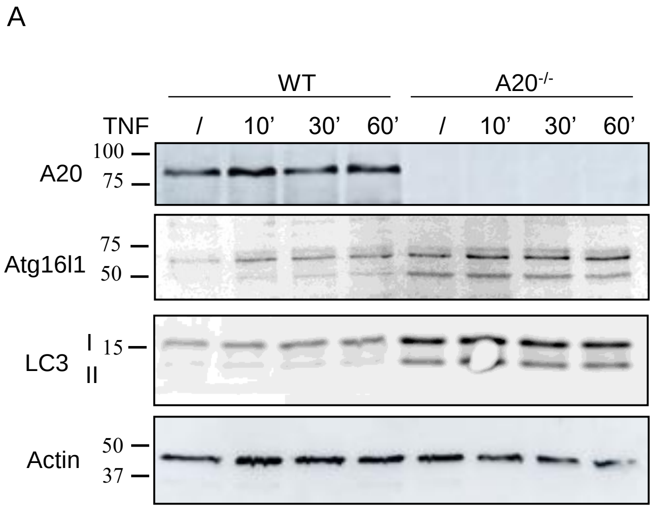

Atg16L1/actin	0,091	0,076	0,106	0,091	0,127	0,226	0,214	0,191
LC3-I/actin	0,538	0,574	0,592	0,779	0,593	0,730	0,745	0,639
LC3-II/actin	0,194	0,251	0,277	0,266	0,408	0,629	0,638	0,608

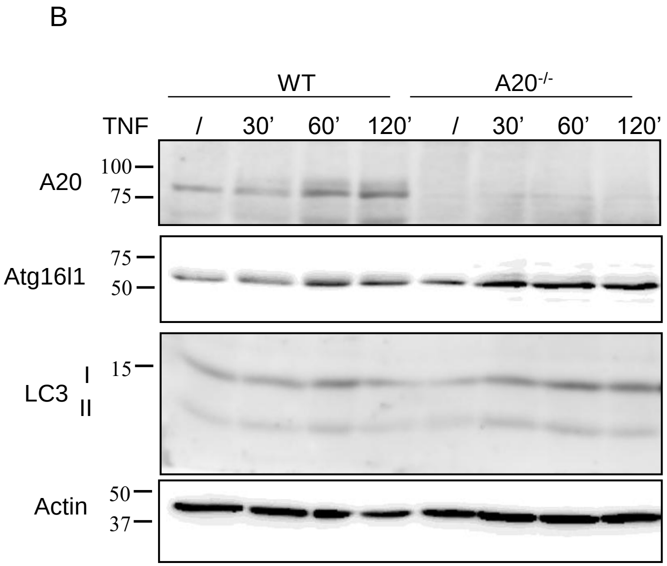

Atg16L1/actin	0,144	0,204	0,436	0,401	0,198	0,448	0,476	0,355
LC3-I/actin	0,150	0,254	0,479	0,246	0,148	0,256	0,359	0,336
LC3-II/actin	0,157	0,110	0,181	0,109	0,111	0,151	0,144	0,091

Supplementary Figure 4. A20 deficiency increases Atg16L1 expression and LC3-II expression levels. (A) Immortalized MEFs were stimulated with 1000 IU/ml of recombinant murine TNF for indicated time points. Data representative of 5 independent experiments. **(B)** Small intestinal organoids were stimulated with 10 ng/ml recombinant TNF for indicated time points. Data representative of 3 independent experiments.

Supplementary Figure 5

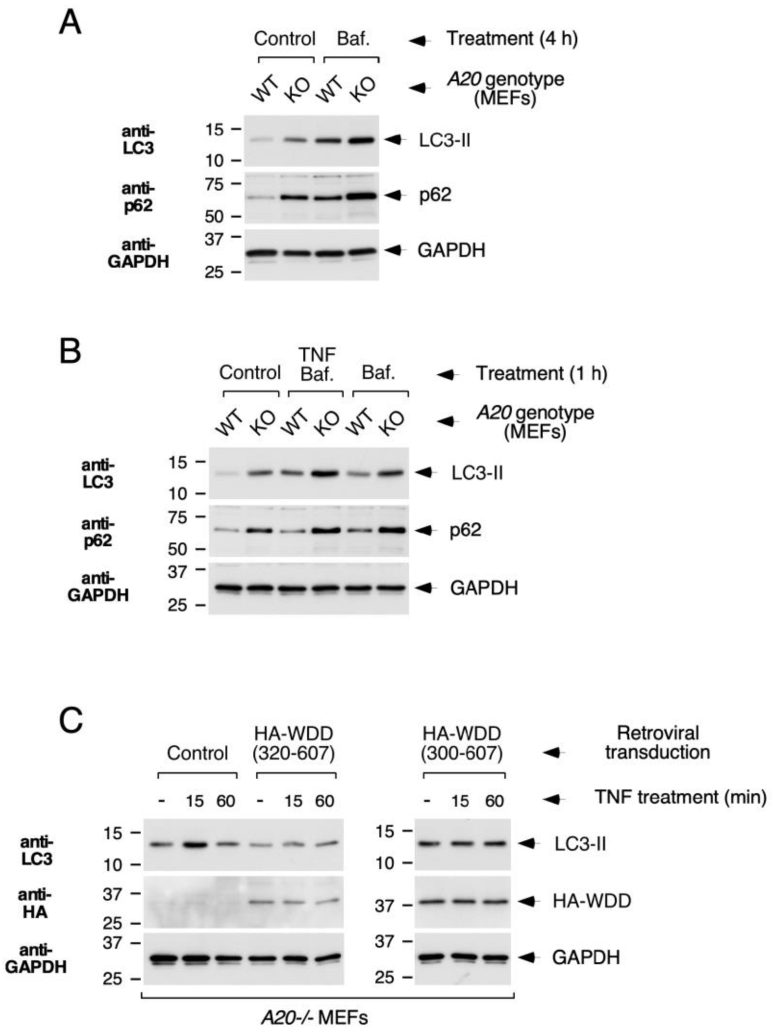

Supplementary Figure 5. (A-B) Increased autophagic flux in A20-deficient cells, both basally (A) and upon TNF treatment (B). Wild-type and A20-deficient MEFs were treated with bafilomycin (Baf., 75 nM) and/or TNF (30 ng/ml) for the indicated times. Cells were then lysed and the resulting total cell lysates were processed for Western-blotting with the shown antibodies. **(C)** Expression of the WDD dominantly inhibits LC3 lipidation induced by TNF treatment in A20-deficient MEFs. A20-/- cells were transduced with retroviral constructs expressing the indicated constructs of the WDD and then subjected to TNF treatment (30 ng/ml) for the indicated times. Cells were lysed and the corresponding total cell lysates subjected to Western-blotting with the indicated antibodies.

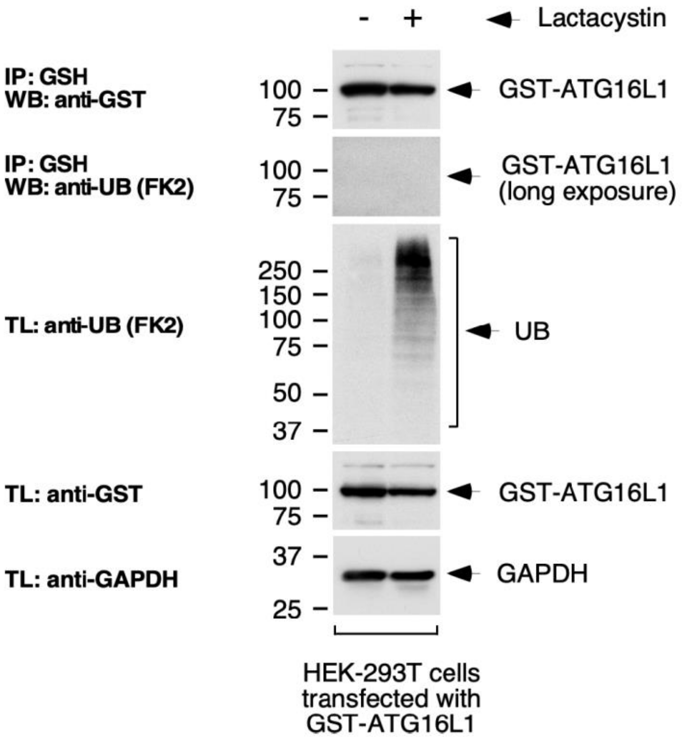

Supplementary Figure 6. Transfected GST-ATG16L1 is not ubiquitinated nor stabilized by the proteasome inhibitor lactacystin. HEK-293T cells were transfected with GST-ATG16L1 and treated with lactacystin (10 uM) for the last 12 h of culture. Cells were lysed and GST-ATG16L1 immunoprecipitated with agarose-GSH beads. The resulting immunoprecipitates and total lysates were processed for Western-blotting using the indicated antibodies.

Supplementary Figure 7

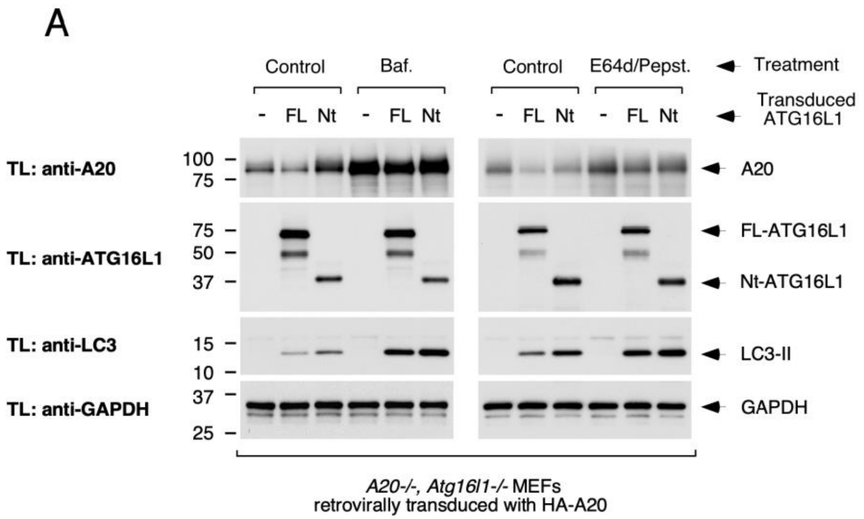

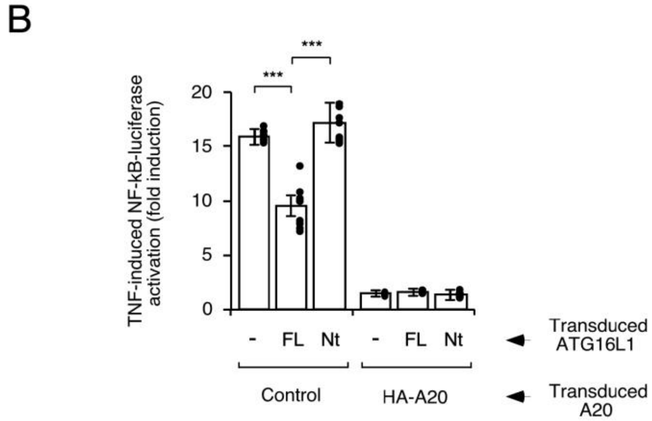

Supplementary Figure 7. (A) Lysosomal inhibition normalizes A20 expression levels in A20/Atg16L1-double deficient MEFs restored with HA-A20 and different ATG16L1 constructs. The indicated cellular strains were treated with bafilomycin (Baf.; 75 nM for 8 h) or E64d/pepstatin (10 ug/ml each for 12 h) and lysed for Western-blotting with the shown antibodies. **(B) ATG16L1 represses NF-κB activation in A20-deficient MEFs.** The indicated cell lines were transduced with a retroviral construct harboring an NF-κB-luciferase reporter cassette, treated with TNF (30 ng/ml, 4 h) and lysed to measure luciferase activity. Shown are average values and the corresponding standard deviations of fold-induction figures obtained from triplicate experimental points (Student’s t-test; (***) $p < 0,001$).

Supplementary Figure 8

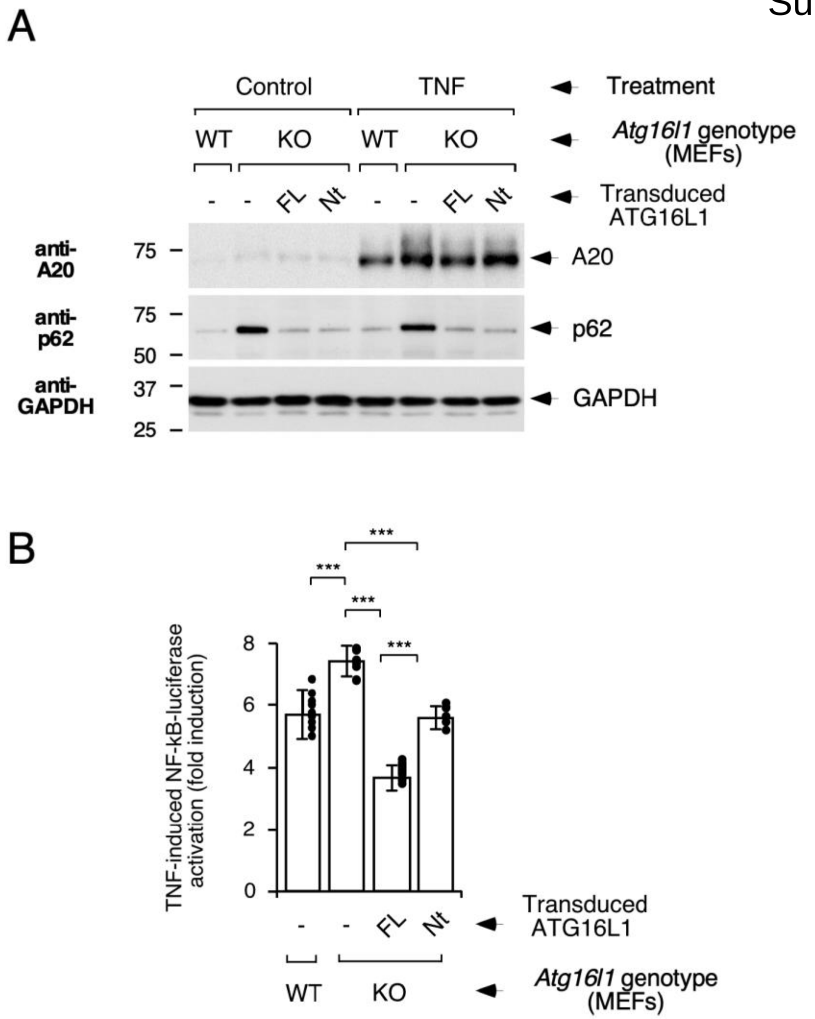

Supplementary Figure 8. (A) Expression of endogenous A20 is regulated by ATG16L1. The indicated wild-type or Atg16L1-deficient MEFs (control (-) or reconstituted with the shown versions of ATG16L1 (full-length: FL; N-terminal domain (1-299): Nt)) were subjected to TNF treatment (30 ng/ml) for 1 h, as shown. Cells were then lysed and processed for Western-blotting with the indicated antibodies. **(B) ATG16L1 regulates NF-κB activation in response to TNF.** The same MEFs shown in A were transduced with a retroviral construct harboring an NF-κB-luciferase reporter cassette, treated with TNF (30 ng/ml, 4 h) and lysed to measure luciferase activity. Shown are average values and the corresponding standard deviations of fold-induction figures obtained from triplicate experimental points (Student’s t-test; (***) p < 0,001).

Supplementary Figure 9

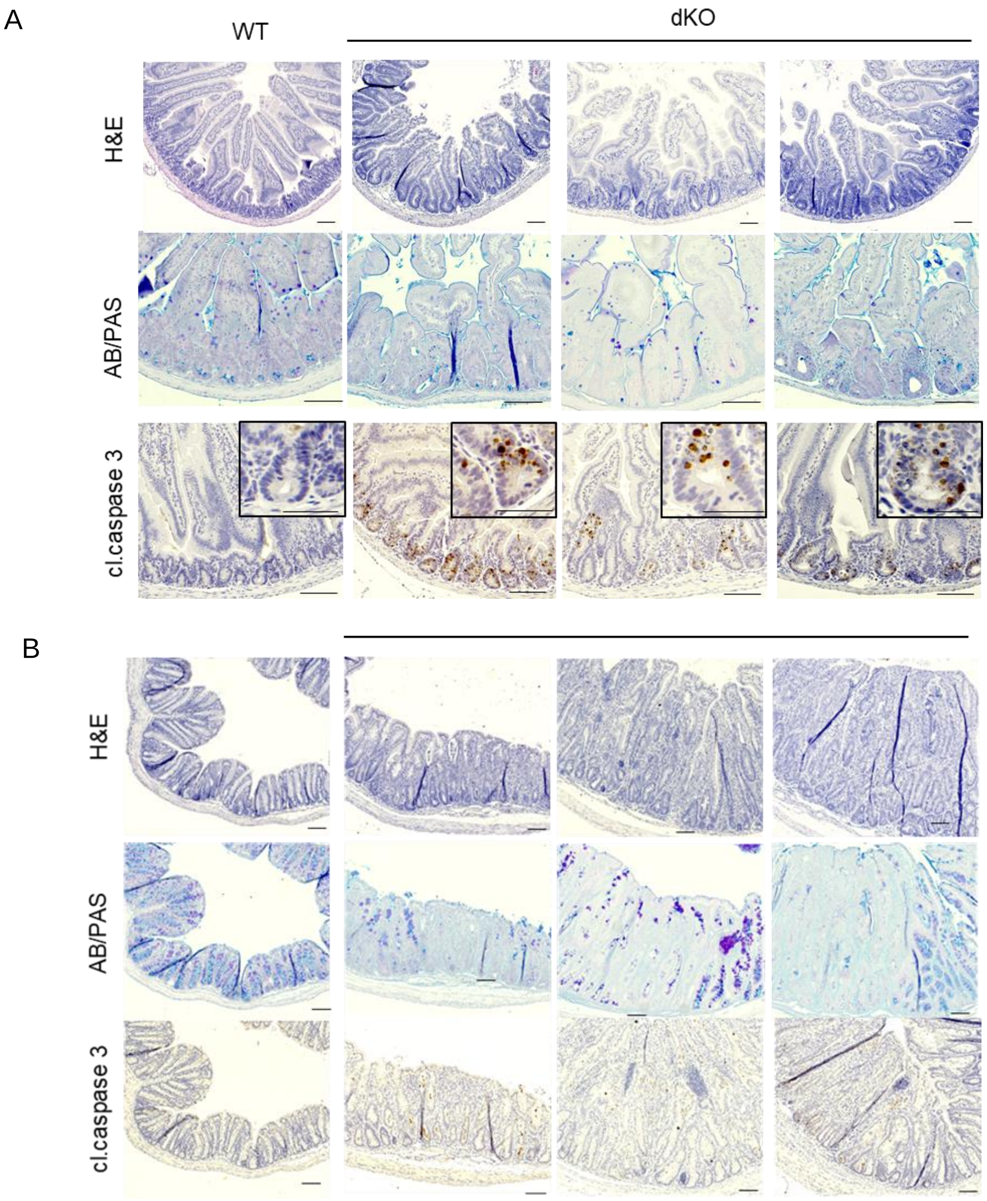

Supplementary Figure 9. (A-B) Hematoxylin-eosin (H&E), AB/PAS and cleaved caspase-3 staining on sections of the proximal small intestinal (A) and colon (B) of three 3-4 week old control (WT) and dKO mice, showing cell death and loss of secretory cells. Scale bar, 100 µm; 50 µm zoom-in panel.

Supplementary Figure 10

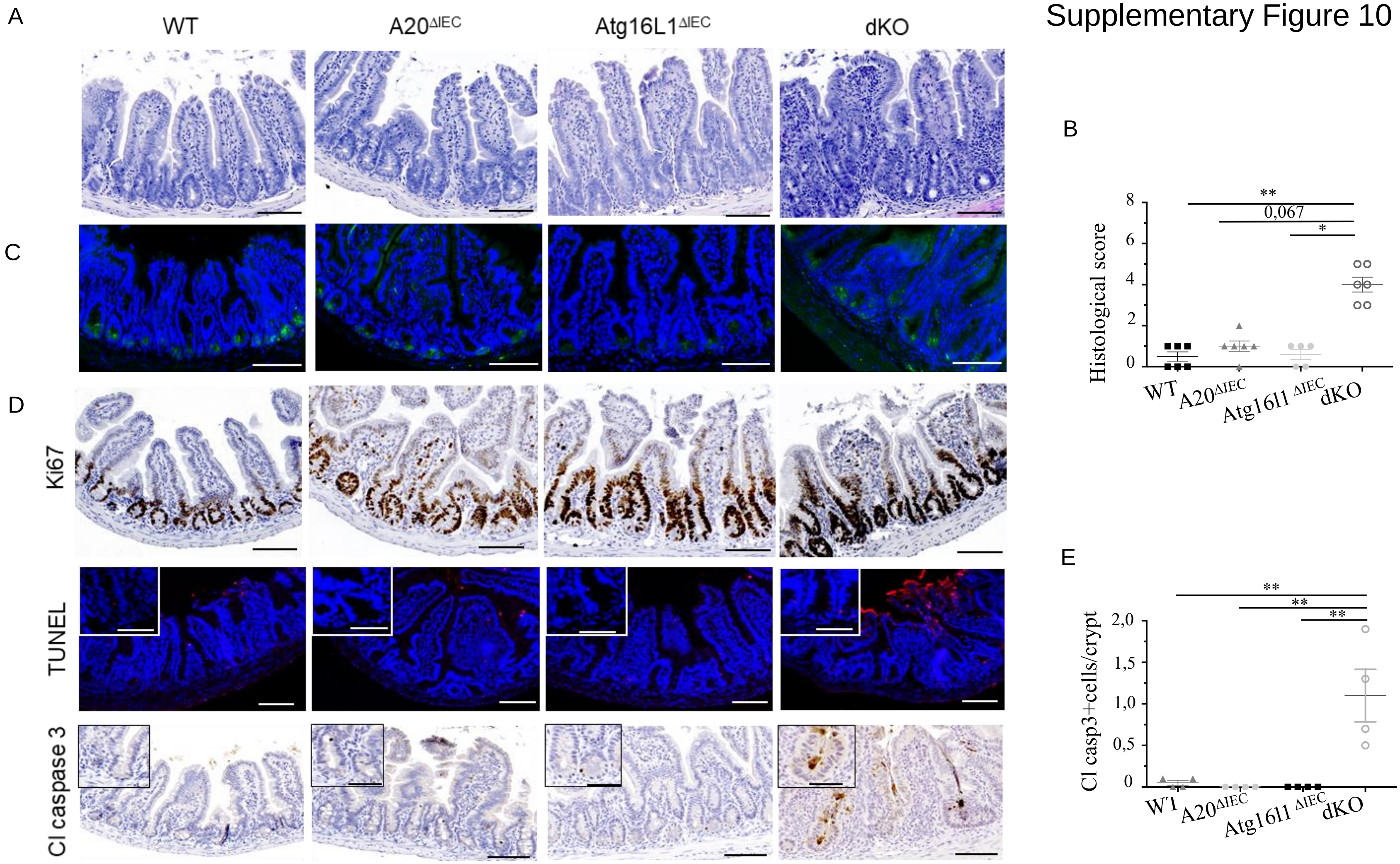

Supplementary Figure 10. (A) Hematoxylin-eosin (H&E) staining of distal small intestinal sections of 20 week old control (WT), A20$^{\Delta IEC}$, Atg16l1$^{\Delta IEC}$ and dKO mice (A; scale bar, 100µm). **(B)** Histological scoring of small intestinal sections from WT, A20$^{\Delta IEC}$, Atg16l1$^{\Delta IEC}$ and dKO mice of distal small intestine. Each symbol represents one mouse. *, $p < 0,05$. **(C)** Immunofluorescent staining of distal small intestinal sections using an antibody recognizing lysozyme in intracellular granules of Paneth cells (green) in WT, A20Δ^{IEC}, Atg16l1$^{\Delta IEC}$ and dKO mice. Cell nuclei were counterstained with DAPI (blue). Scale bar, 100 µm. **(D)** Immunostaining for Ki67, TUNEL (red) and cleaved caspase 3 on sections from the distal small intestine of WT, A20$^{\Delta IEC}$, Atg16l1$^{\Delta IEC}$ and dKO mice. Images representative of n=5 mice per genotype. Cell nuclei were counterstained with DAPI. Scale bars, 100 µm; inserts 50 µm. **(E)** Quantification of cleaved caspase 3-positive cells in sections from the distal small intestine of WT, A20$^{\Delta IEC}$, Atg16l1$^{\Delta IEC}$ and dKO mice.

Supplementary Figure 11

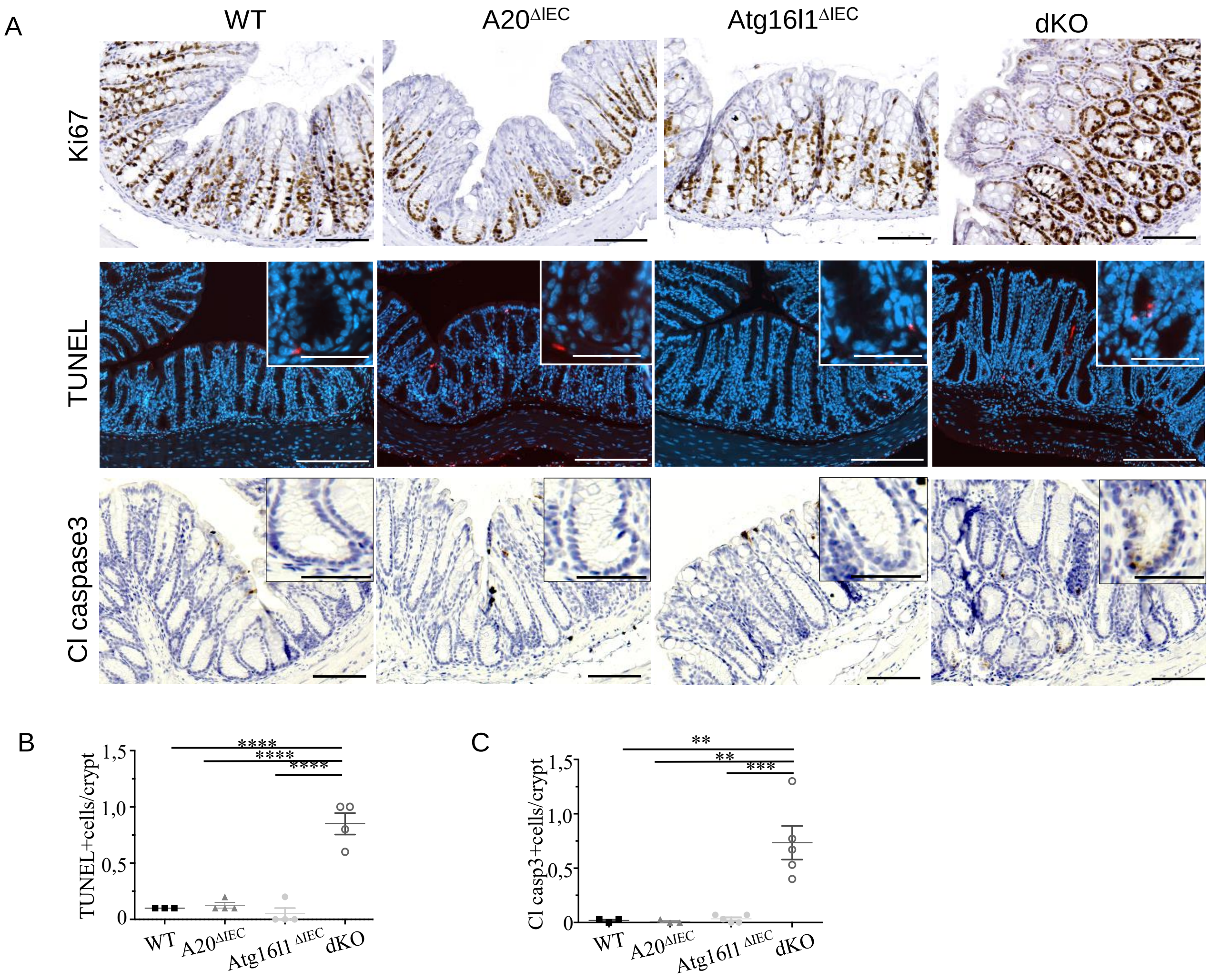

Supplementary Figure 11. (A) Immunostaining for Ki67, TUNEL (red) and cleaved caspase 3 on colon sections from WT, A20$^{\Delta IEC}$, Atg16l1$^{\Delta IEC}$ and dKO mice. Images representative of n=3-5 mice per genotype. Cell nuclei were counterstained with DAPI. Scale bars, bright-field 100µm; fluorescence 200µm; inserts 50µm **(B-C)** Quantification of TUNEL (B) and cleaved caspase 3-(C) positive cells in colon sections from WT, A20$^{\Delta IEC}$, Atg16l1$^{\Delta IEC}$ and dKO mice.

Supplementary Figure 12

A

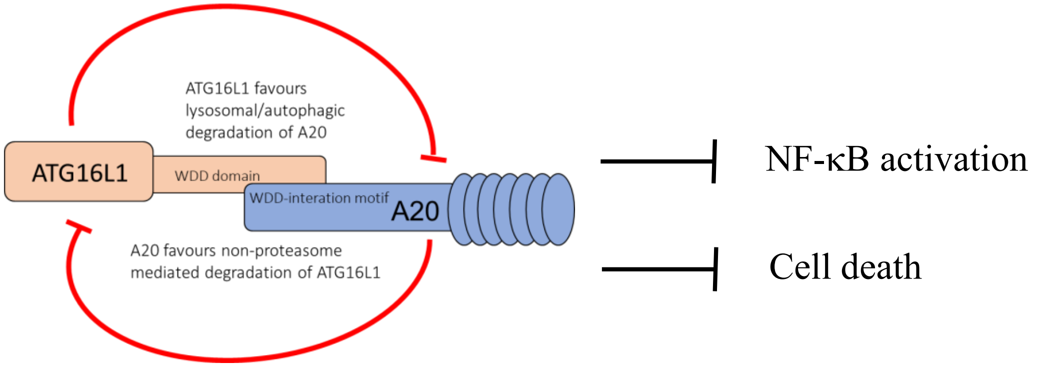

B

	WT	*A20-/-*	*Atg16l1-/-*	*A20/Atg16l1-/-*
**A20 and Atg16l1 protein levels**	Balanced homeostatic levels	Atg16l1 stabilization	A20 stabilization	Absent
**Autophagy**	Balanced	Increased autophagy (LC3-II↑)	Defective autophagy	Defective autophagy
**NF-κB activation**	Balanced	Counterbalanced	Increased, despite A20 stabilisation	Increased
***In vivo* pathological cell death**	Prevented	Prevented in steady state (sensitive to TNF induced death)	Prevented in steady state (sensitive to TNF induced death)	Spontaneous enterocyte cell death

Supplementary Figure 12. Summarizing model demonstrating A20-Atg16L1 cross-regulation to control intestinal homeostasis. Induction of Atg16l1 in A20-deficient cells promotes unconventional autophagy, enhanced p62 expression and reduced NF-κB activation that could keep under control the increased levels of NF-κB caused by absence of A20. Conversely, A20 upregulation in cells lacking Atg16l1 correlates with higher levels of p62, increased NF-κB activation and protection against cell death. Combined A20 and Atg16l1 deficiency promotes NF-κB-dependent inflammation and cell death inducing spontaneous intestinal pathology.

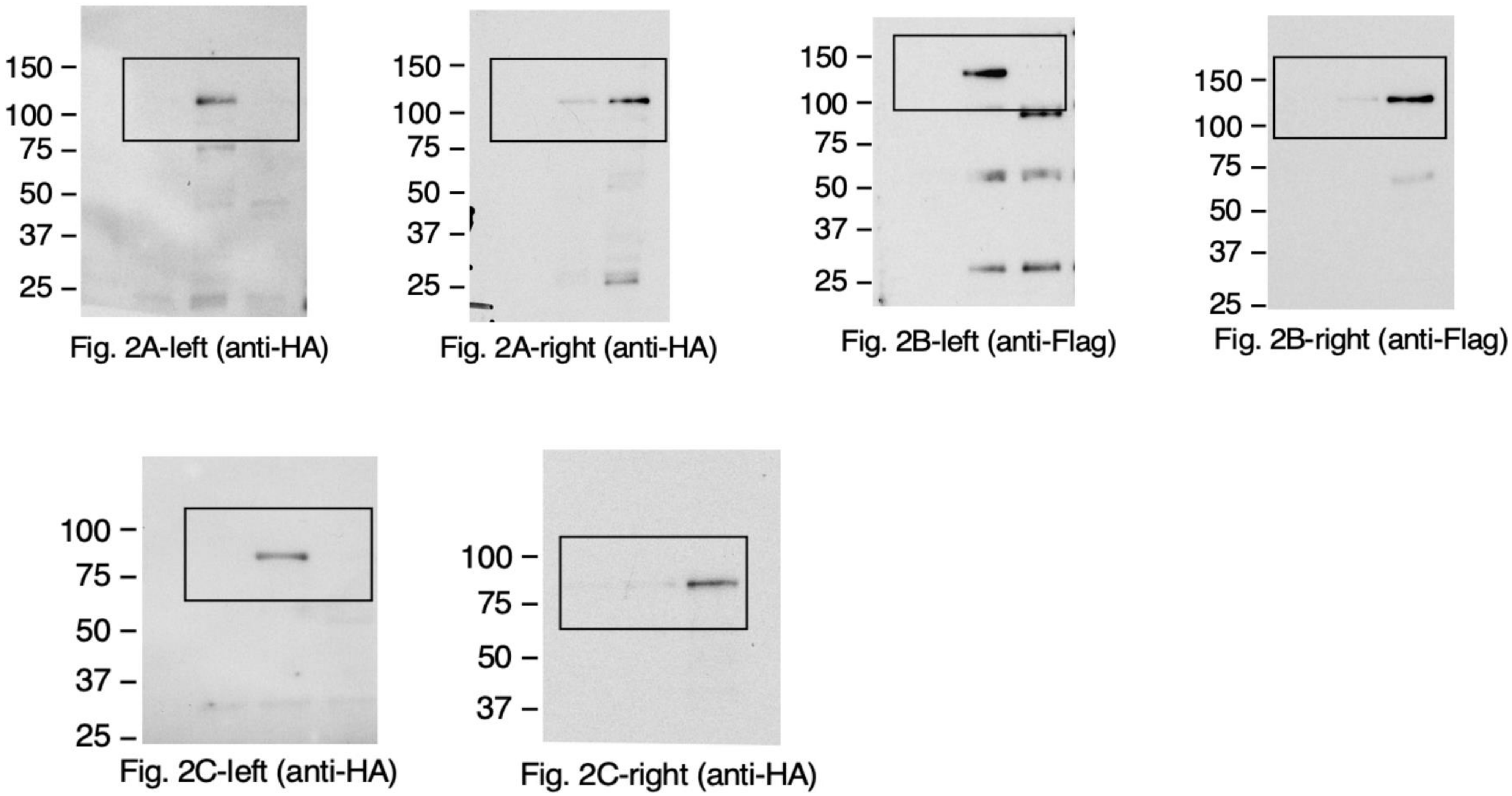

Supplementary Figure 13. Uncropped scans of all Western blots

Fig.2

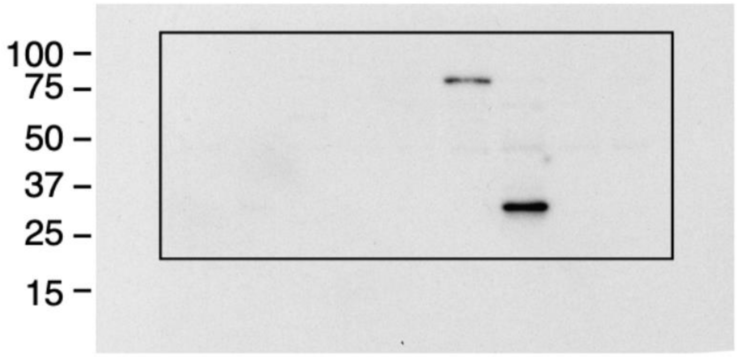

Fig. 3A (anti-HA)

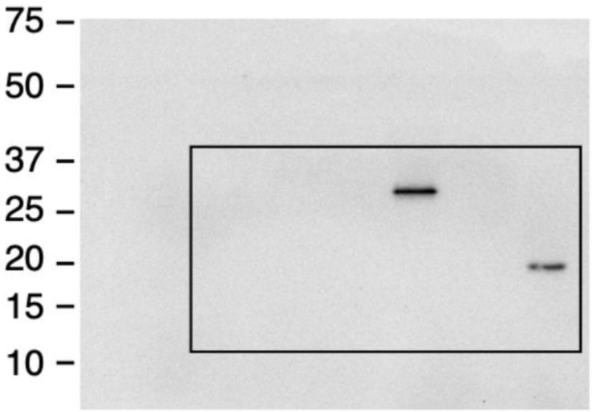

Fig. 3B (anti-HA)

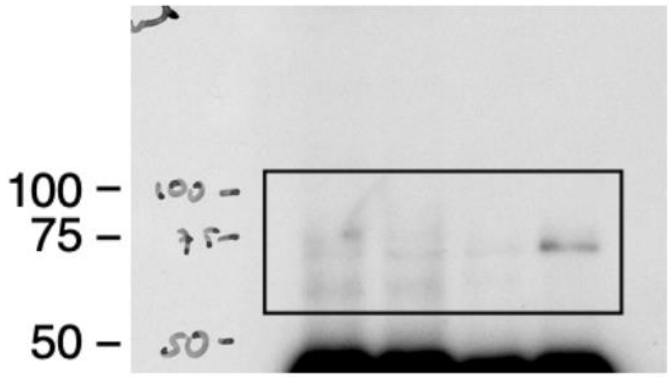

Fig. 3C-left (anti-Atg16l1)

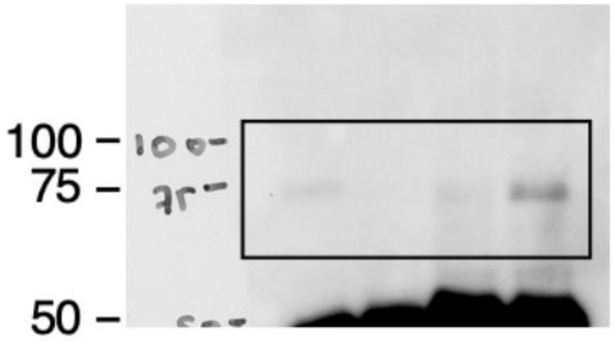

Fig. 3C-right (anti-Atg16l1)

Fig.3

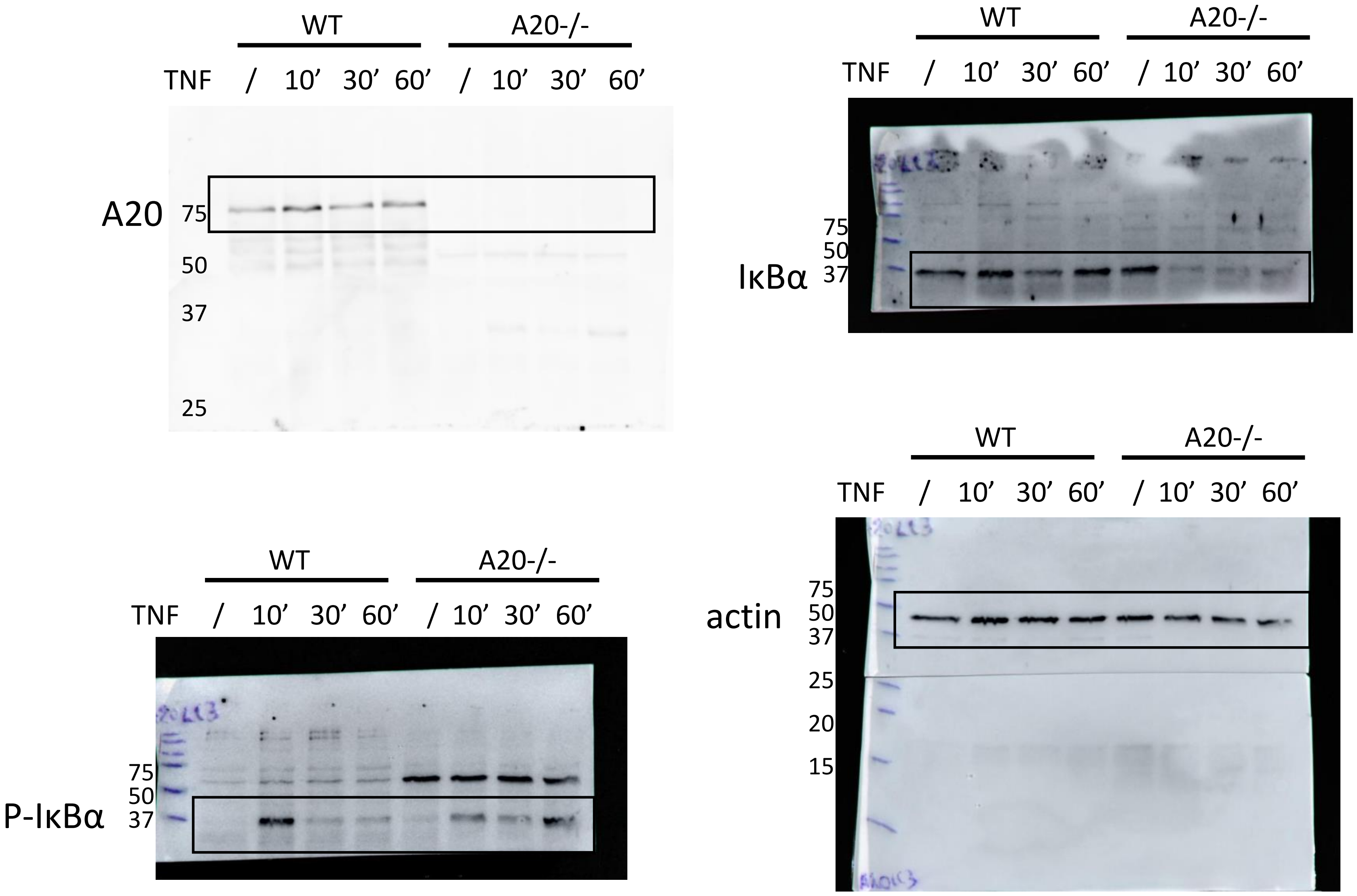

Fig.4 A top panel

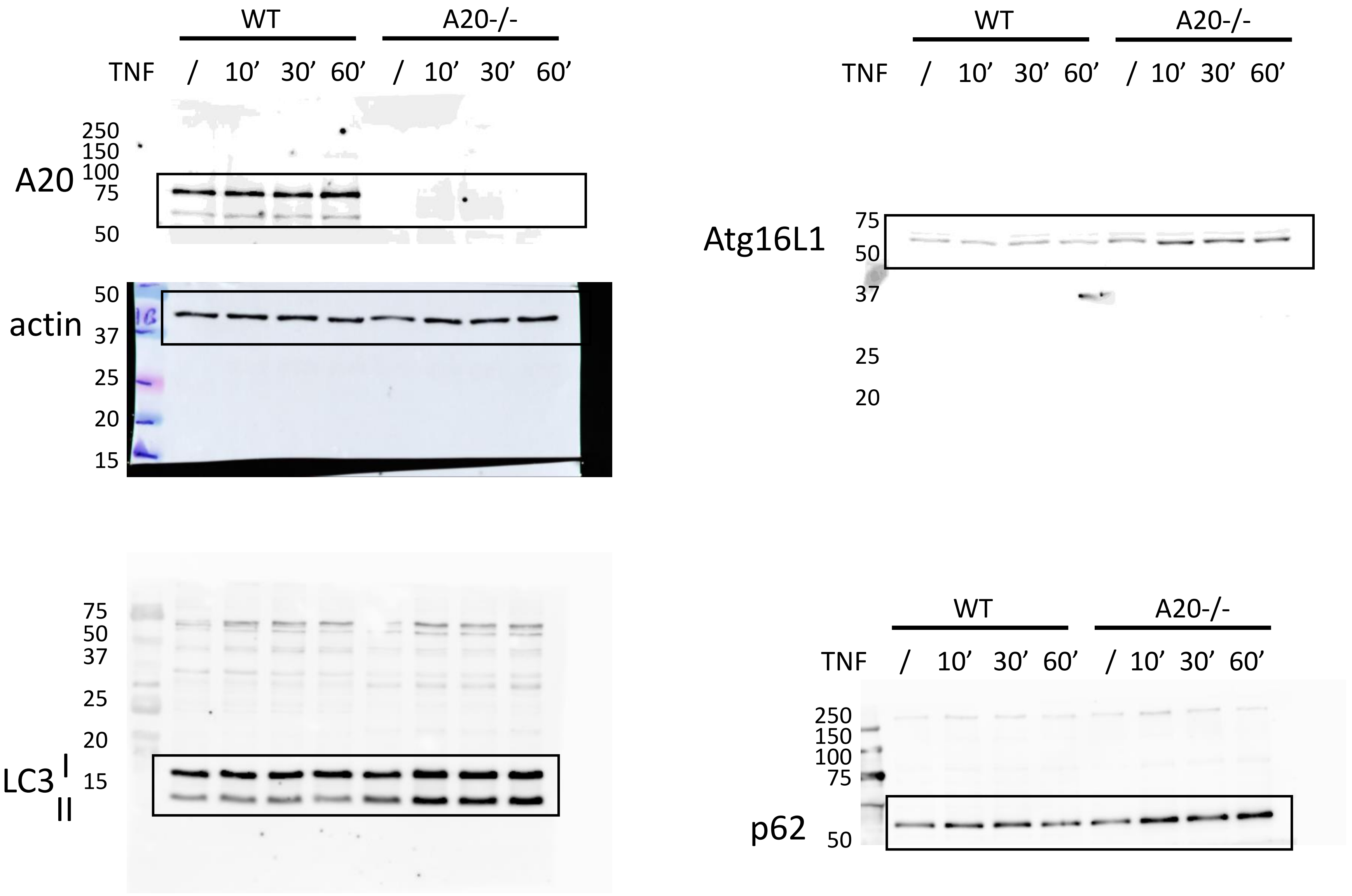

Fig.4 A bottom panel

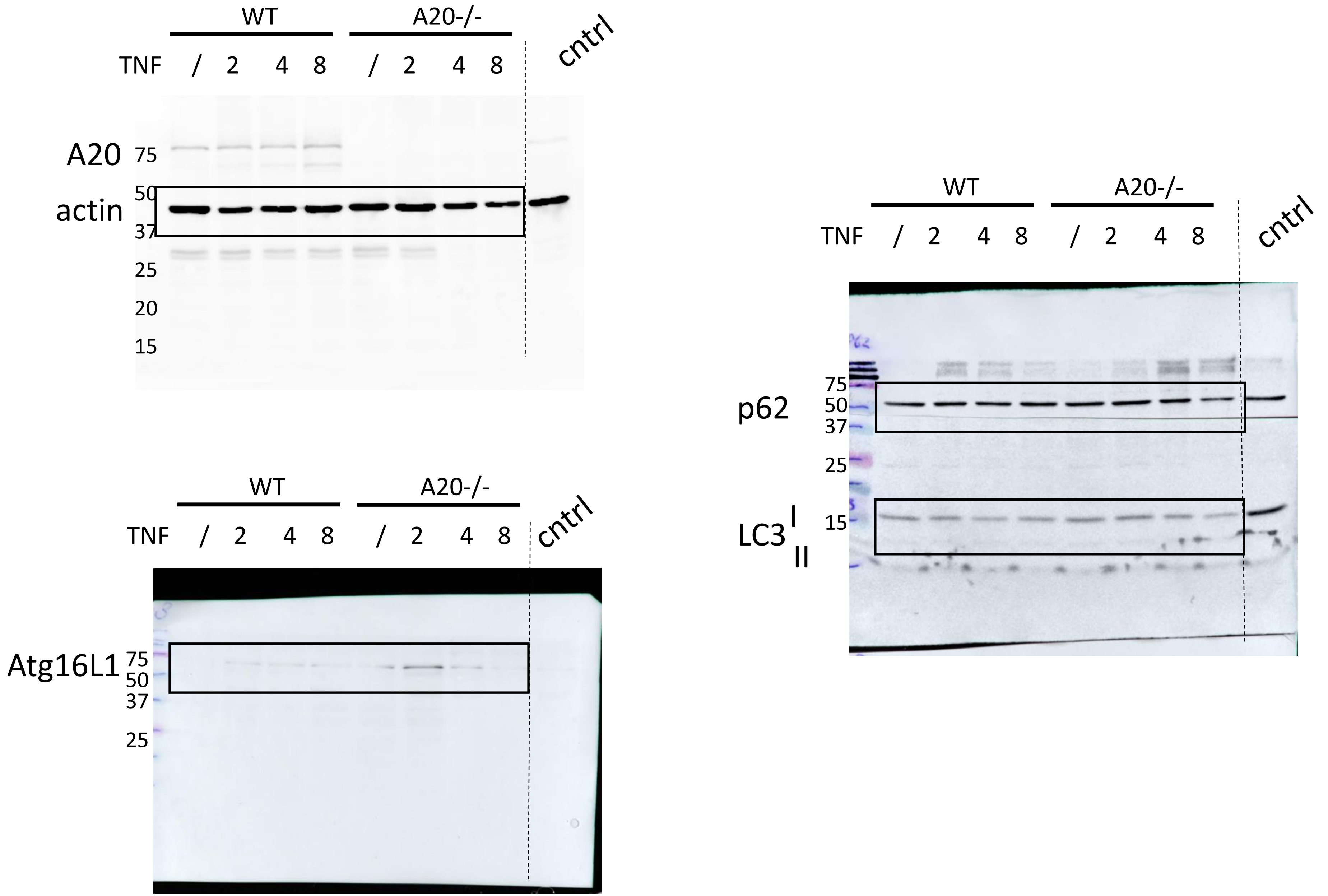
WT
A20-/-
TNF
/
2
4
8
cntrl
A20
actin
75
50
37
25
20
15
Atg16L1
p62
LC3
I
II

Fig.4 B

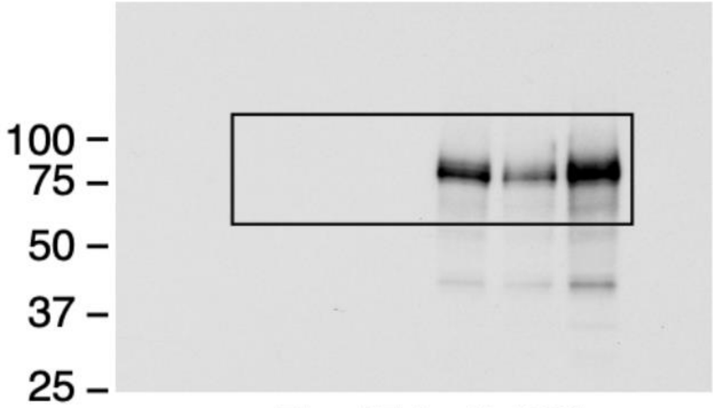

Fig. 4E (anti-A20)

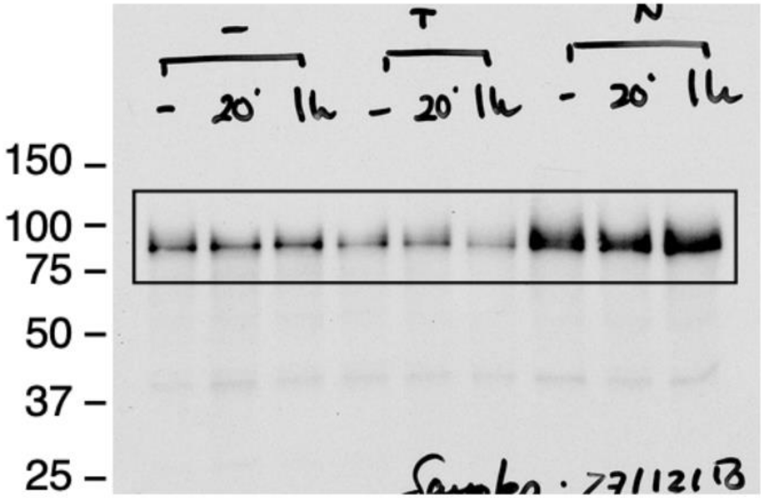

Fig. 4F (anti-A20)

Fig.4

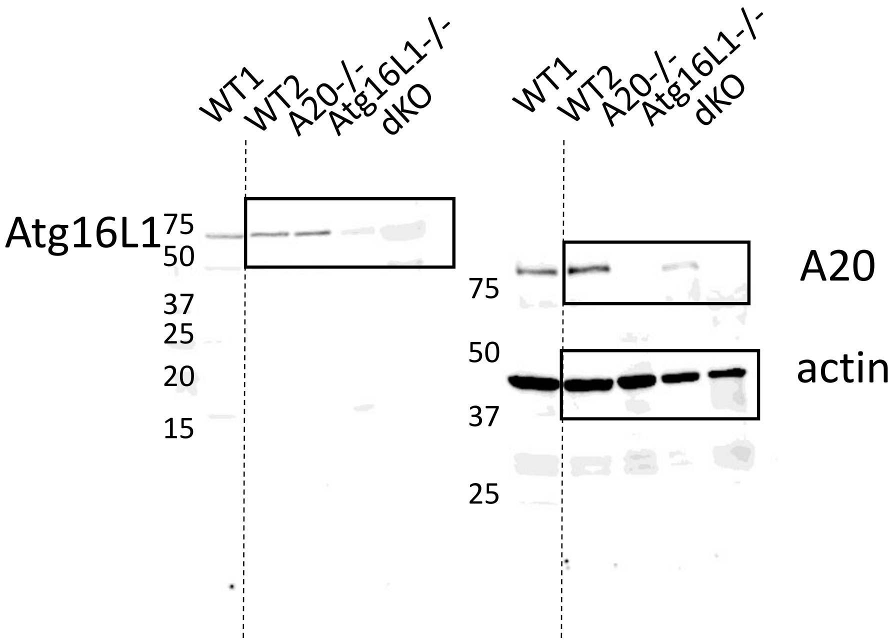

WT1
WT2
A20-/-
Atg16L1-/-
dKO
Atg16L1
75
50
37
25
20
15
WT1
WT2
A20-/-
Atg16L1-/-
dKO
A20
actin
75
50
37
25

Fig.5 A

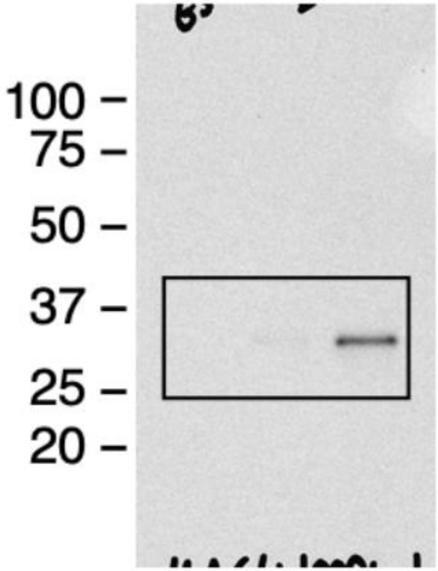

Suppl. Fig. 2A (anti-HA)

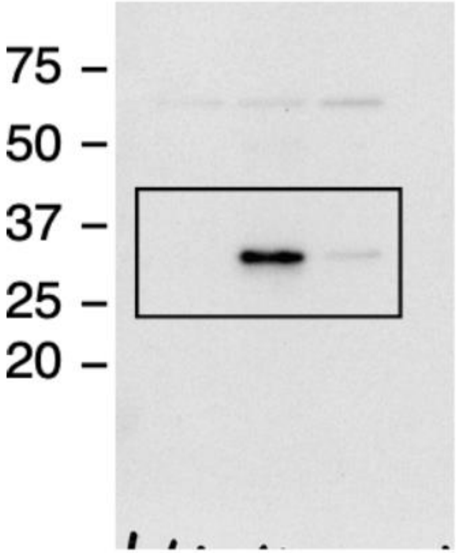

Suppl. Fig. 2B
(anti-HA)

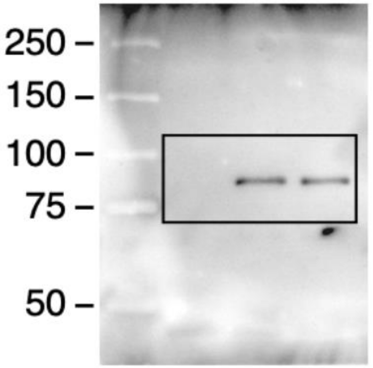

Suppl. Fig. 3A
(anti-HA)

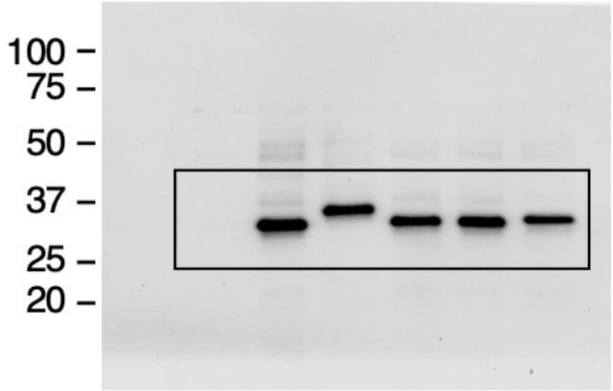

Suppl. Fig. 3B-left
(anti-HA)

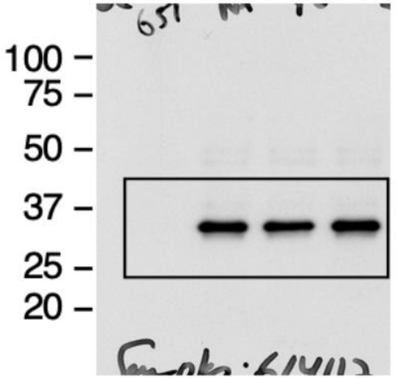

Suppl. Fig. 3B-center
(anti-HA)

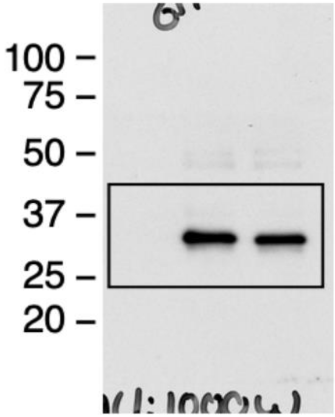

Suppl. Fig. 3B-right
(anti-HA)

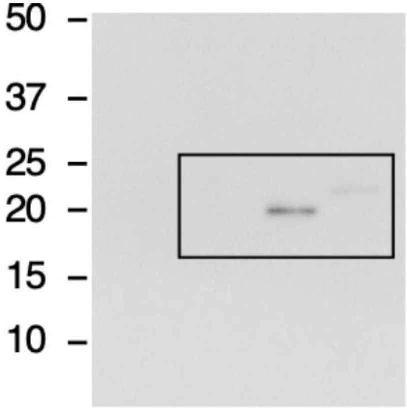

Suppl. Fig. 3C
(anti-HA)

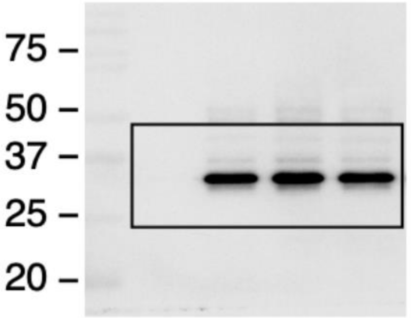

Suppl. Fig. 3D
(anti-HA)

Suppl. Fig. 3

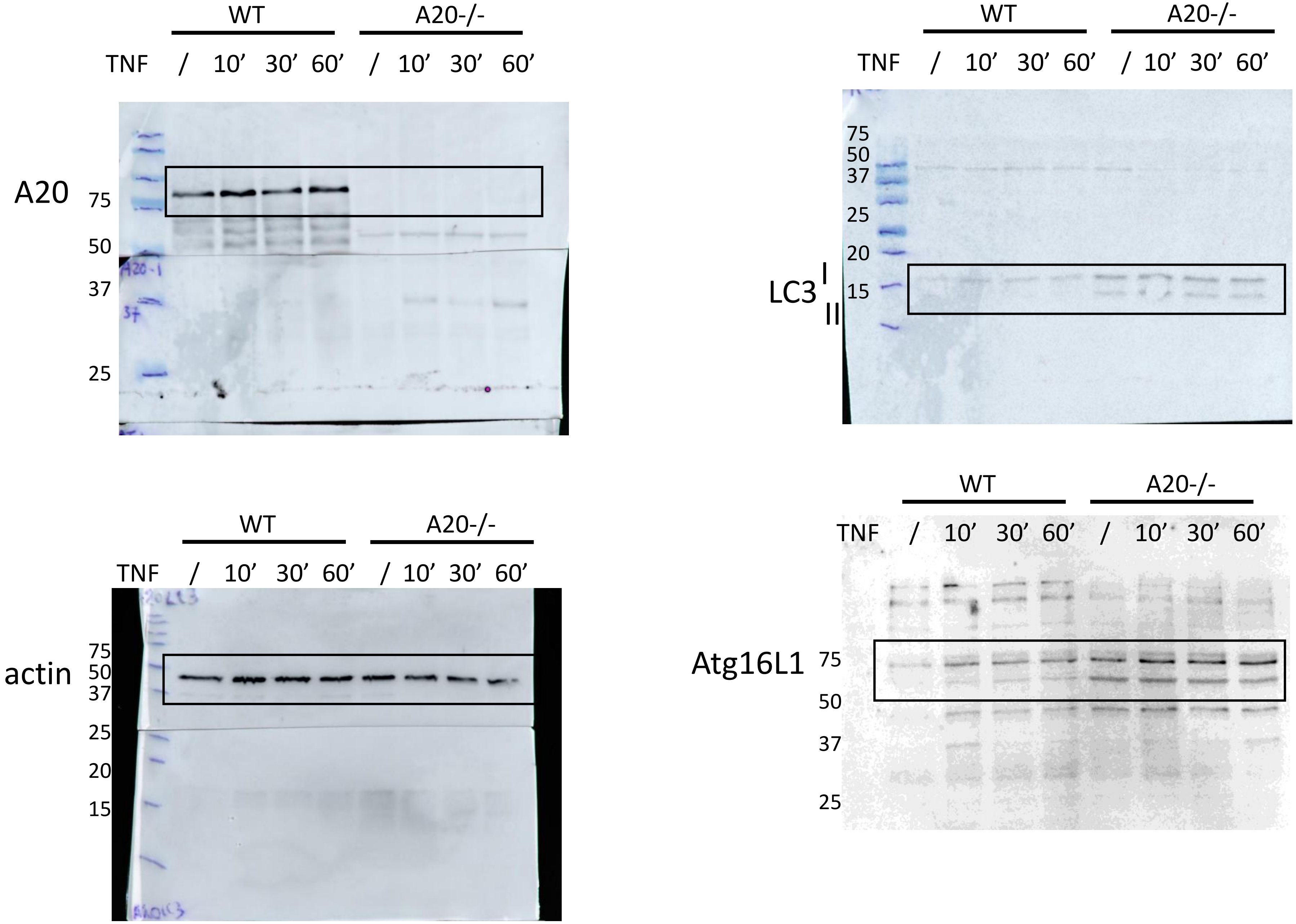
WT
A20-/-
TNF / 10’ 30’ 60’ / 10’ 30’ 60’
A20
75
50
37
25
LC3
I
II
75
50
37
25
20
15
actin
75
50
37
25
20
15
Atg16L1
75
50
37
25

Sup. Fig.4 A

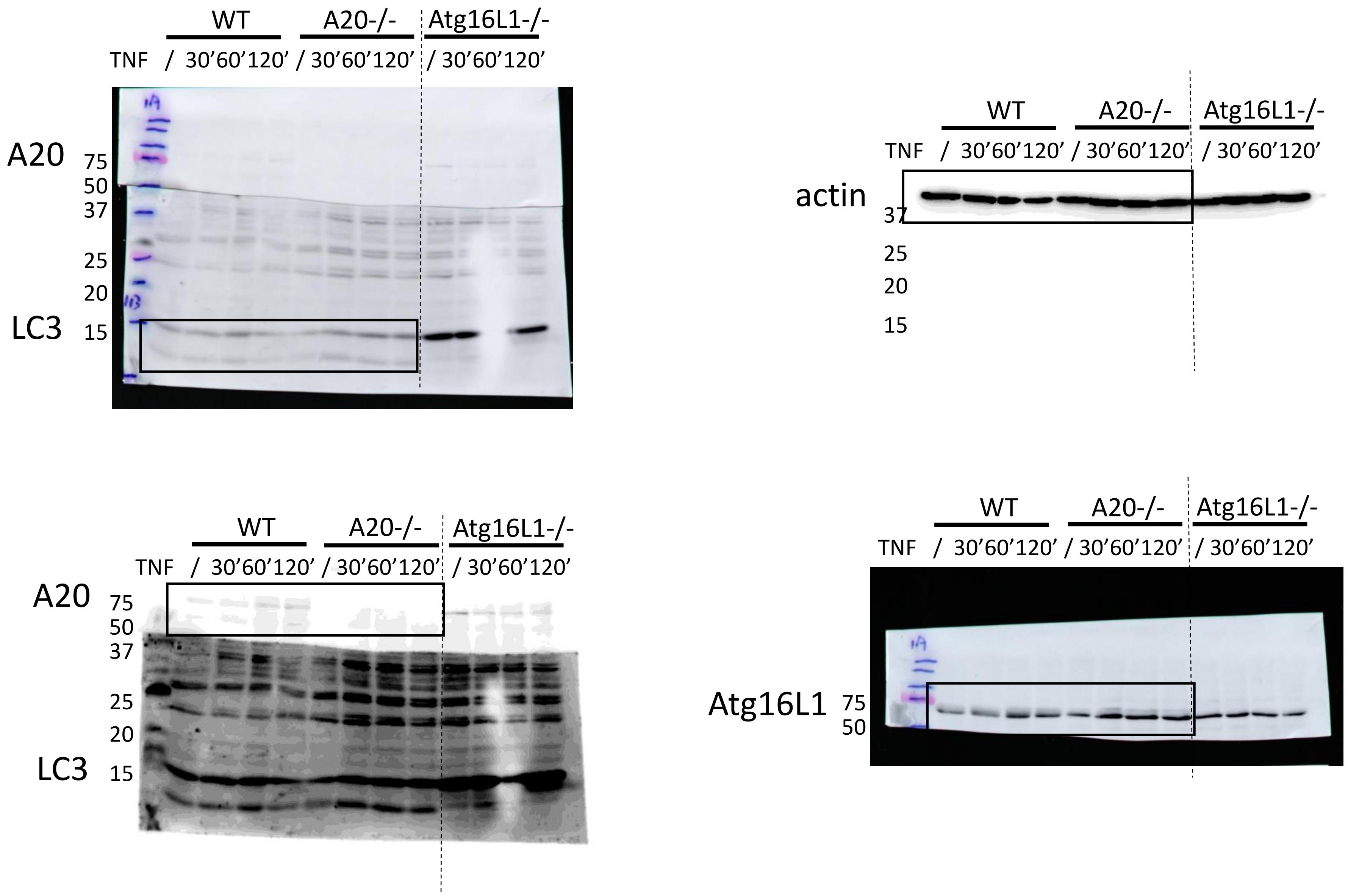
WT
A20-/-
Atg16L1-/-
TNF / 30’60’120’ / 30’60’120’ / 30’60’120’
A20
75
50
37
25
20
LC3
15
actin
WT
A20-/-
Atg16L1-/-
TNF / 30’60’120’ / 30’60’120’ / 30’60’120’
37
25
20
15
WT
A20-/-
Atg16L1-/-
TNF / 30’60’120’ / 30’60’120’ / 30’60’120’
A20
75
50
37
25
20
LC3
15
WT
A20-/-
Atg16L1-/-
TNF / 30’60’120’ / 30’60’120’ / 30’60’120’
Atg16L1
75
50

Sup. Fig.4 B

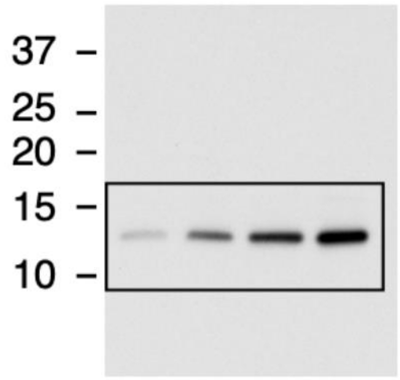

Suppl. Fig. 5A
(anti-LC3)

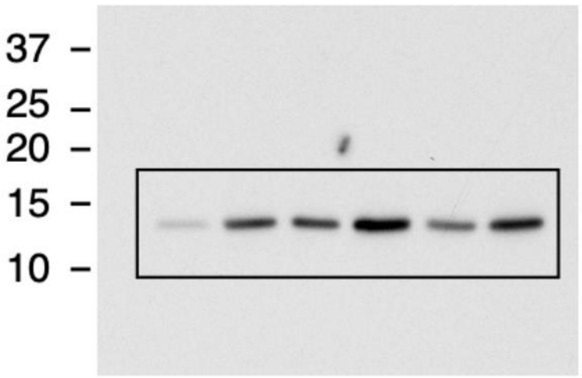

Suppl. Fig. 5B
(anti-LC3)

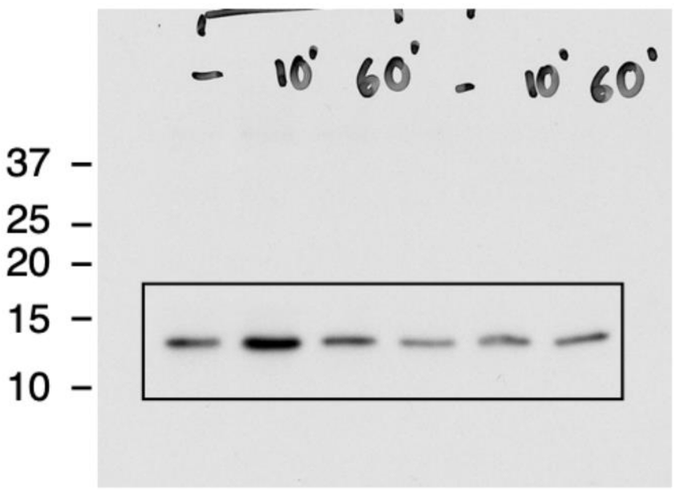

Suppl. Fig. 5C-left
(anti-LC3)

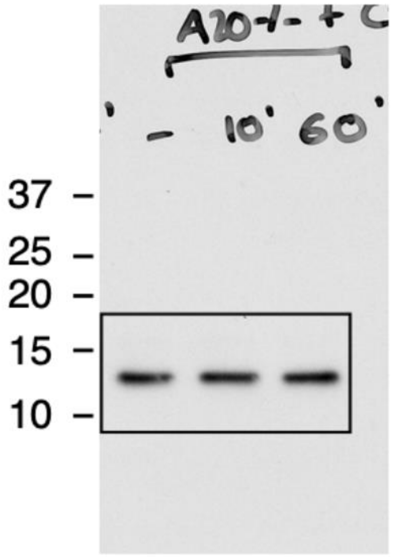

Suppl. Fig. 5C-right
(anti-LC3)

Suppl. Fig. 5

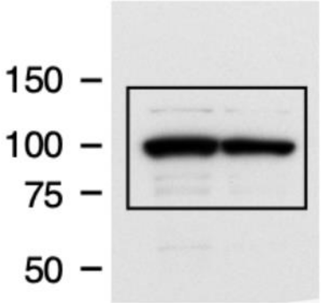

Suppl. Fig. 6-top
(anti-GST)

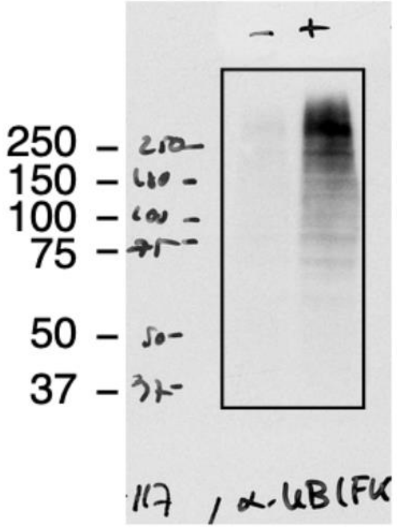

Suppl. Fig. 6-center
(anti-UB (FK2))

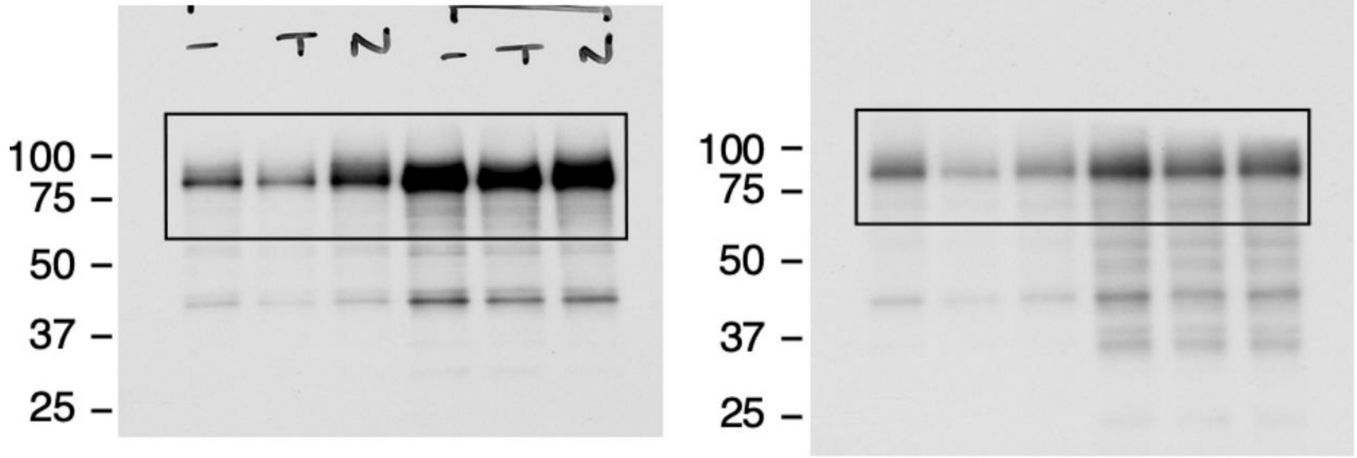

Suppl. Fig. 7A-left
(anti-A20)

Suppl. Fig. 7A-right
(anti-A20)

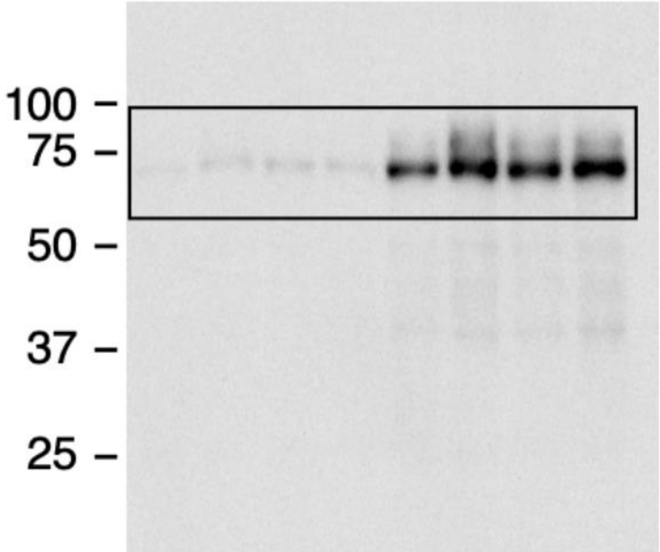

Suppl. Fig. 8A
(anti-A20)

Supplementary Table 1. Oligonucleotides for PCR

A20-Not-1-fw	5’ gggcccgcggccgcaatggctgaacaagtccttcctc 3’
A20-Xho-stop-263-rev	5’ cccgggctcgagtttacagggtcaccaagggtacaaaatgatggc 3’
A20-Not-264-fw	5’ gggcccgcggccgcaaaggacagtgggcctgaaatccgagctgttc 3’
A20-Not-357-fw	5’ gggcccgcggccgcacaggaaaacagcgagcaggggaggagag 3’
A20-Xho-stop-790-rev	5’ cccgggctcgagtttagccatacatctgcttgaactgaaagcattc 3’
A20-Not-92-fw	5’ gggcccgcggccgcacttgtggcgctgaaaacgaacggtgacgg 3’
A20-Xho-stop-91-rev	5’ cccgggctcgagtttacttccggacttctcgacaccagttgagtttc 3’

Sequences of the oligonucleotides used to generate A20 constructs by PCR.

Supplementary Table 2. Oligonucleotides for site-directed mutagenesis

A20-F127A,L130A-TOP	5’ gtactgaggaaggcgctggccagcacggccaaggaaacagacacacgcaac 3’
A20-F127A,L130A-BOTT	5’ gttgcgtgtgtctgtttccttggccgtgctggccagcgccttcctcagtac 3’
A20-W168A,L171A-TOP	5’ cggaactggaatgatgaagccgacaatgctatcaaaatggcttccacag 3’
A20-W168A,L171A-BOTT	5’ ctgtggaagccattttgatagcattgtcggcttcatcattccagttccg 3’
A20-Y188A,L191A-TOP	5’ gcccgaagtggacttcaggccaactcagccgaagaaatacacatatttg 3’
A20-Y188A,L191A-BOTT	5’ caaatatgtgtatttcttcggctgagttggcctgaagtccacttcgggc 3’
A20-F224A,L227A-TOP	5’ gtttggaatcaggttccaatgccgcccctgcgaaagtgggtggaatttacttg 3’
A20-F224A,L227A-BOTT	5’ caagtaaattccacccactttcgcaggggcggcattggaacctgattccaaac 3’
A20-Y233A,L236A-TOP	5’ ctttgaaagtgggtggaattgccttgcctgcccactggcctgcccaggaatg 3’
A20-Y233A,L236A-BOTT	5’ cattcctgggcaggccagtgggcaggcaaggcaattccacccactttcaaag 3’
A20-L227A,Y233A,L236A-TOP	5’ ctgcgaaagtgggtggaattgccttgcctgcccactggcctgcccaggaatg 3’
A20-L227A,Y233A,L236A-BOTT	5’ cattcctgggcaggccagtgggcaggcaaggcaattccacccactttcgcag 3’
A20-Y246A,V249A-TOP	5’ gcccaggaatgctacagagcccccattgctctcggctatgacagccatc 3’
A20-Y246A,V249A-BOTT	5’ gatggctgtcatagccgagagcaatgggggctctgtagcattcctgggc 3’
A20-F257A,L260A-TOP	5’ ggctatgacagccatcatgctgtacccgcggtgaccctgaaggacagtg 3’
A20-F257A,L260A-BOTT	5’ cactgtccttcagggtcaccgcgggtacagcatgatggctgtcatagcc 3’
A20-A125V-TOP	5’ cttggtactgaggaaggtgctgttcagcacgctcaag 3’
A20-A125V-BOTT	5’ cttgagcgtgctgaacagcaccttcctcagtaccaag 3’
A20-F127C-TOP	5’ ctgaggaaggcgctgtgcagcacgctcaaggaaac 3’
A20-F127C-BOTT	5’ gtttccttgagcgtgctgcacagcgccttcctcag 3’

Sequences of the oligonucleotides used to generate A20 WDD-binding motif mutants by site-directed mutagenesis.
