# Supplementary material for: Physical and functional interaction between A20 and ATG16L1-WD40 domain in the control of intestinal homeostasis
Source: Nat Commun. 2019 Apr 23;10:1834. doi: 10.1038/s41467-019-09667-z (PMC6478926; doi:10.1038/s41467-019-09667-z)
Supplement: Supplementary file 3 — Reporting Summary [file 41467_2019_9667_MOESM3_ESM.pdf]

nature research

Corresponding author(s): *Double-blind peer review submissions: write DBPR and your manuscript number here instead of author names.*

Last updated by author(s): *YYYY-MM-DD*

Reporting Summary

Nature Research wishes to improve the reproducibility of the work that we publish. This form provides structure for consistency and transparency in reporting. For further information on Nature Research policies, see Authors & Referees and the Editorial Policy Checklist.

Statistics

For all statistical analyses, confirm that the following items are present in the figure legend, table legend, main text, or Methods section.

n/a	Confirmed	
☐	☒	The exact sample size (*n*) for each experimental group/condition, given as a discrete number and unit of measurement
☐	☒	A statement on whether measurements were taken from distinct samples or whether the same sample was measured repeatedly
☐	☒	The statistical test(s) used AND whether they are one- or two-sided<br>*Only common tests should be described solely by name; describe more complex techniques in the Methods section.*
☐	☒	A description of all covariates tested
☐	☒	A description of any assumptions or corrections, such as tests of normality and adjustment for multiple comparisons
☐	☒	A full description of the statistical parameters including central tendency (e.g. means) or other basic estimates (e.g. regression coefficient) AND variation (e.g. standard deviation) or associated estimates of uncertainty (e.g. confidence intervals)
☐	☒	For null hypothesis testing, the test statistic (e.g. *F*, *t*, *r*) with confidence intervals, effect sizes, degrees of freedom and *P* value noted<br>*Give P values as exact values whenever suitable.*
☒	☐	For Bayesian analysis, information on the choice of priors and Markov chain Monte Carlo settings
☒	☐	For hierarchical and complex designs, identification of the appropriate level for tests and full reporting of outcomes
☒	☐	Estimates of effect sizes (e.g. Cohen's *d*, Pearson's *r*), indicating how they were calculated

Our web collection on statistics for biologists contains articles on many of the points above.

Software and code

Policy information about availability of computer code

Data collection	No software was used for data collection
Data analysis	Statistical analysis was performed using GraphPad prism V7

For manuscripts utilizing custom algorithms or software that are central to the research but not yet described in published literature, software must be made available to editors/reviewers. We strongly encourage code deposition in a community repository (e.g. GitHub). See the Nature Research guidelines for submitting code & software for further information.

Data

Policy information about availability of data

All manuscripts must include a data availability statement. This statement should provide the following information, where applicable:

- Accession codes, unique identifiers, or web links for publicly available datasets
- A list of figures that have associated raw data
- A description of any restrictions on data availability

There are no restrictions on data availability

Field-specific reporting

Please select the one below that is the best fit for your research. If you are not sure, read the appropriate sections before making your selection.

☒ Life sciences ☐ Behavioural & social sciences ☐ Ecological, evolutionary & environmental sciences

For a reference copy of the document with all sections, see nature.com/documents/nr-reporting-summary-flat.pdf

Life sciences study design

All studies must disclose on these points even when the disclosure is negative.

Sample size	Based on our previous experience, to evaluate the spontaneous phenotype in A20/Atg16l1 dKO animals we aimed to analyze 5-10 animals per group. Experiments were performed at least 2-3 times to confirm reproducibility.
Data exclusions	No data were excluded from the experiments
Replication	All experiments were performed at least 2-3 times
Randomization	No specific method of randomization had been used to select animals
Blinding	No blinding was done

Reporting for specific materials, systems and methods

We require information from authors about some types of materials, experimental systems and methods used in many studies. Here, indicate whether each material, system or method listed is relevant to your study. If you are not sure if a list item applies to your research, read the appropriate section before selecting a response.

Materials & experimental systems

n/a	Involved in the study	
☐	☒	Antibodies
☐	☒	Eukaryotic cell lines
☒	☐	Palaeontology
☐	☒	Animals and other organisms
☒	☐	Human research participants
☒	☐	Clinical data

Methods

n/a	Involved in the study	
☒	☐	ChIP-seq
☒	☐	Flow cytometry
☒	☐	MRI-based neuroimaging

Antibodies

Antibodies used	rabbit anti-Ki67, dilution 1/1000, Cell Signaling 12202; rabbit anti-lysozyme, dilution 1/700, Dako A0099; anti-cleaved caspase-3, 1/700 dilution, Cell Signaling 9661; mouse anti-A20 (Santa Cruz sc-166692, 1:1000), rabbit anti-Atg16lL1 (Cell Signaling 8089, 1:1000); rabbit anti-LC3 (MBL PM036, 1:1000); rabbit anti-p62 (MBL PM045, 1:2000), goat anti-IkBa (Santa Cruz sc-371-G, 1:1000); mouse anti-P-IkBa (Cell Signaling 9246, 1:1000); mouse anti-actin (MP Biomedicals 8691002, 1:10000); mouse anti-HA (BioLegend 901501, 1:1000); mouse anti-Flag (Sigma F1804, 1:1000); rabbit anti-GST (Cell Signaling 2622, 1:1000); mouse anti-Tubulin (Sigma T4026, 1:40000); mouse anti-GAPDH (Abcam Ab8245, 1:10000); mouse anti-Atg16l1 (MBL 150-3, 1:2000); mouse anti-LC3 (MBL M186-3, 1:2000); mouse anti-UB (FK2; Millipore 4-263, 1:1000).
Validation	All antibodies have been properly validated

Eukaryotic cell lines

Policy information about cell lines

Cell line source(s)	primary MEF cells and organoids generated from the mouse lines; HEK293T cells
Authentication	*Describe the authentication procedures for each cell line used OR declare that none of the cell lines used were authenticated.*
Mycoplasma contamination	Mycoplasma negative
Commonly misidentified lines (See ICLAC register)	N.A.

Animals and other organisms

Policy information about studies involving animals; ARRIVE guidelines recommended for reporting animal research

Laboratory animals	C57BL/6J male and female mice were used in these studies.
Wild animals	This study did not involve wild animals

Field-collected samples	This study did not involve field-collected samples
Ethics oversight	Animal protocols were approved by the ethics committee of Ghent University.

Note that full information on the approval of the study protocol must also be provided in the manuscript.
