# Supplementary material for: Physical and functional interaction between A20 and ATG16L1-WD40 domain in the control of intestinal homeostasis
Source: Nat Commun. 2019 Apr 23;10:1834. doi: 10.1038/s41467-019-09667-z (PMC6478926; doi:10.1038/s41467-019-09667-z)
Supplement: Supplementary file 4 — Description of Additional Supplementary Files [file 41467_2019_9667_MOESM4_ESM.pdf]

Description of Additional Supplementary Files

Supplementary Data 1:

Complete lists of Uniprot entries and associated mass spectrometry data of the proteins identified in the different cellular systems. Lists are arranged according to the cell lines and conditions used for the proteomics approach: JAR cells, unactivated THP-1 cells and THP-1 cells activated with LPS (5 µg/ml) and PMA (50 ng/ml) for 24 h.

Supplementary Data 2:

Curated lists of Uniprot entries identified in the proteomics assay. Entries shown in Supplementary Data 1 were curated to remove non-human and redundant references, manually substitute obsolete and unreviewed entries for reviewed ones, and select those present in less than 10% (41) of the unspecific assays included in the Crapome database (411).

Supplementary Data 3:

Venn analysis of the identified proteins. Proteins included in Supplementary Data 2 were subjected to a Venn algorithm to establish the degree of redundancy of the proteins identified in the three experimental systems.

Supplementary Data 4:

Unified list of identified proteins classified according to their functional annotation. Entries shown in Supplementary Data 2 were classified according to their annotation in the Function [CC] and/or Gene Ontology (biological process) fields (Uniprot) as involved in different cellular processes, including inflammation/ innate immunity, apoptosis/cell death, adhesion, autophagy, DNA replication/ damage/repair, immune response, cell signaling or intracellular trafficking.
